# Robust Classification of Intramuscular EMG Signals to Aid the Diagnosis of Neuromuscular Disorders

**DOI:** 10.1109/OJEMB.2020.3017130

**Published:** 2020-08-17

**Authors:** Shobha Jose, S. Thomas George, M. S. P. Subathra, Vikram Shenoy Handiru, Poornaselvan Kittu Jeevanandam, Umberto Amato, Easter Selvan Suviseshamuthu

**Affiliations:** School of Engineering and TechnologyKarunya Institute of Technology and Sciences 121735 Coimbatore 641-114 India; Center for Mobility and Rehabilitation Engineering ResearchKessler Foundation 158368 West Orange NJ 07052 USA; Data Business GroupAccenture Bridgewater NJ 08807 USA; Istituto di Scienze Applicate e Sistemi Intelligenti ‘Eduardo Caianiello,’ Consiglio Nazionale delle Ricerche 519169 80131 Napoli Italy

**Keywords:** Fractal dimension, intramuscular electromyography, lifting wavelet transform, local binary pattern, majority vote, multilayer perceptron neural network, neuromuscular disorders

## Abstract

*Goal:* This article presents the design and validation of an accurate automatic diagnostic system to classify intramuscular EMG (iEMG) signals into healthy, myopathy, or neuropathy categories to aid the diagnosis of neuromuscular diseases. *Methods:* First, an iEMG signal is decimated to produce a set of “disjoint” downsampled signals, which are decomposed by the lifting wavelet transform (LWT). The Higuchi's fractal dimensions (FDs) of LWT coefficients in the subbands are computed. The FDs of LWT subband coefficients are fused with one-dimensional local binary pattern derived from each downsampled signal. Next, a multilayer perceptron neural network (MLPNN) determines the class labels of downsampled signals. Finally, the sequence of class labels is fed to the Boyer-Moore majority vote (BMMV) algorithm, which assigns a class to every iEMG signal. *Results:* The MLPNN-BMMV classifier was experimented with 250 iEMG signals belonging to three categories. The performance of the classifier was validated in comparison with state-of-the-art approaches. The MLPNN-BMMV has resulted in impressive performance measures (%) using a 10-fold cross-validation—accuracy = $99.87\pm 0.25$, sensitivity (normal) = $99.97\pm 0.13$, sensitivity (myopathy) = $99.68\pm 0.95$, sensitivity (neuropathy) = $99.76\pm 0.66$, specificity (normal) = $99.72\pm 0.61$, specificity (myopathy) = $99.98\pm 0.10$, and specificity (neuropathy) = $99.96\pm 0.14$—surpassing the existing approaches. *Conclusions:* A future research direction is to validate the classifier performance with diverse iEMG datasets, which would lead to the design of an affordable real-time expert system for neuromuscular disorder diagnosis.

## I. Introduction

Electromyography (EMG) is the recording of electrical activities produced by skeletal muscles with noninvasive or invasive methods. Surface EMG (sEMG) is recorded using surface electrodes placed on the skin above the muscle for prosthetic control and rehabilitation purposes [1]–[3]. Whereas, intramuscular EMG (iEMG) is collected using a monopolar or concentric needle electrode inserted through the skin into the muscle tissue. Neuromuscular disorders are known to alter the morphology and physiology of the basic component of the peripheral nervous system called the motor unit (MU). Since stimulation of motor neurons induces contraction or shortening of the muscle fibers, any damage to motor neurons affects muscle function [1], [4]. The extracellular needle EMG records the motor unit action potential (MUAP), the analysis of which provides crucial information for the diagnosis of neuromuscular disorders [1]. Healthy MUAPs have only two to four phases and are smaller in amplitude. By contrast, neurogenic disorders are characterized by long-duration, high-amplitude, and polyphasic MUAPs; in myopathy, MUAPs are smaller in amplitude, polyphasic, and of shorter duration [1], [5]. Furthermore, a frequency content shift toward low and high frequencies is observed in the iEMG signal collected from subjects suffering from neuropathy and myopathy, respectively [6], [7]. Traditionally, MUAPs are examined by the visual and audio characteristics of iEMG signals. However, this practice has been proven effective only to detect some disorders and remains inadequate to explain less apparent deviations or assorted patterns of abnormalities [8]. Therefore, several computer-aided EMG classification algorithms have been developed over the last two decades to accurately classify the iEMG data for reliably diagnosing neuromuscular abnormalities [8].

Typically, a computer-based EMG classifier first extracts features from the raw EMG signals and then employs a classification algorithm to discriminate these features. The selection of an optimal feature set is pivotal for improving the classification accuracy [9], [10]. Therefore, diverse feature sets were explored by various researchers: time domain features [5], [11], [12]; frequency domain features [13]–[16]; and time-frequency (TF) domain features [17]–[25]. An alternate approach is to combine features from different domains [8], [26]–[29].

In like manner, several classifiers were attempted to enhance the iEMG classification performance. Typical examples include artificial neural networks (ANN) [12], [14], [20], [27]–[30]; deep learning algorithm [17]; neuro-fuzzy system [8], [13]; support vector machine (SVM) [7], [18], [26], [31], [32]; $k$-nearest neighbor (KNN) [19], [22], [24], [33]; machine learning [11]; quadratic classifier [21]; and random forest decision tree [23], [34]. Moreover, iEMG signals were often preprocessed prior to classification, e.g., [5], [15], [16].

We propose a combination of multilayer perceptron neural network (MLPNN) and Boyer-Moore majority vote (BMMV), jointly known as MLPNN-BMMV, which is supplied with fused iEMG feature sets. Each feature vector is generated by concatenating Higuchi's fractal dimensions (FDs) of lifting wavelet transform (LWT) coefficients in the subbands and one-dimensional (1-D) local binary pattern (LBP) of a preprocessed iEMG signal.

The myopathy and neuropathy cause a decrease and increase in the number of functional muscle fibers per MU, respectively (p. 226 of [1]). Consequently, neuromuscular disorders alter the duration, amplitude, and phase of MUAPs, and hence induce changes in the iEMG signal characteristics (see Supp. Material I-A). The FD is a useful metric to quantify various MUAP properties buried in the iEMG signal (e.g., firing rate, MUAP amplitude, waveform phases, etc.) and to track variations in signal structure [35]. Furthermore, FD can distinguish specific states of physiological functions from electrophysiological signals [36] and characterize the complexity of nonstationary and nonlinear iEMG signals [37]. On the other hand, 1-D LBP is sensitive to local changes in a signal. Hence it is proven effective in the diagnosis of Parkinson's disease from gait signals [38], Alzheimer's disease [39] and epilepsy [40] from electroencephalography (EEG) signals, and arrhythmia/atrial fibrillation from electrocardiography (ECG) signals [41], [42]. Besides, it has been applied to distinguish muscle activities and rest from sEMG signals [43]. Recall that neuromuscular diseases alter both MUAP firing rates and configurations [44]. Based on the capability of 1-D LBP to capture the signal morphology in terms of relative changes in amplitude within a neighborhood of an electrophysiological signal, we hypothesize that it can help characterize abnormal iEMG patterns caused by a pathological condition. We therefore fused the FD and 1-D LBP features because they carry complementary information: the FD serves as a global measure of complexity quantifying variations in the signal structure, whereas, the 1-D LBP represents the local signal activity around each data point.

The empirical results from the classification of preprocessed (without downsampling) iEMG signals in Table I reveal that the ANN and a few variants of SVM could lead to a better classification compared to discriminant analysis, decision tree, and ensemble learning. Nevertheless, the classification performance does not heavily rely on the choice of the classifier. Instead, the downsampling plays a pivotal role in enhancing the classifier performance by “artificially” increasing the number of samples. This is because an increase in the sample size improves the classification accuracy by reducing the variance of the estimator, even though the samples are not independent. Furthermore, shorter samples due to downsampling do not interfere with the classifier performance to our advantage.

**TABLE I:** The iEMG Signals Were Classified 25 Times (10-Fold CV) Using a Set of Classifiers Listed in the Left Column. The Average $\mathrm{Ac}$ (%) From 25 Runs Is Reported for Each Classifier. The Best Classifier Under Each Category and the Respective Average $\mathrm{Ac}$ Are Boldfaced.

iEMG Classifier	Average $\mathrm{Ac}$ (%)
**LDA**	${\bf 50.40}$
**FT**	${\bf 73.20}$
Coarse Tree	72.00
Medium Tree	72.40
Ensemble Boosted Tree	75.20
Ensemble Bagged Tree	77.39
**ESD**	${\bf 78.67}$
Ensemble Subspace KNN	66.56
Ensemble Random Undersampling Boosted Tree	78.56
**FKNN**	${\bf 74.80}$
Medium KNN	72.00
Coarse KNN	60.00
Cosine KNN	74.00
Cubic KNN	72.00
Weighted KNN	73.60
Linear SVM	80.59
**QSVM**	${\bf 85.97}$
CSVM	81.97
Fine Gaussian SVM	60.00
Medium Gaussian SVM	77.60
Coarse Gaussian SVM	60.00
MLPNN	86.29
**MLPNN-BMMV**	${\bf 99.87}$

A comparison of performance measures—classification accuracy, sensitivity, and specificity—of MLPNN-BMMV with those of competing approaches is provided in Section III-A. Importantly, the MLPNN-BMMV has contributed to outstanding performance measures—accuracy = $99.87\pm 0.25$, sensitivity (normal) = $99.97\pm 0.13$, sensitivity (myopathy) = $99.68\pm 0.95$, sensitivity (neuropathy) = $99.76\pm 0.66$, specificity (normal) = $99.72\pm 0.61$, specificity (myopathy) = $99.98\pm 0.10$, and specificity (neuropathy) = $99.96\pm 0.14$—surpassing those of state-of-the-art approaches. The novelty of the proposed classifier is four-fold:

1) The LWT has not yet been applied to analyze or classify either sEMG or iEMG signals.
2) FDs of wavelet subband coefficients are introduced to classify iEMG signals.
3) 1-D LBP has been used so far to analyze gait [38], EEG [39], [40], ECG [41], [42], and sEMG signals [43], but not in the context of iEMG classification.
4) MLPNN classifier outputs are refined with BMMV in a unique manner. The majority voting algorithm is conventionally fed with the labels of signal segments, e.g., [5]; whereas, we adopted BMMV to refine the labels of “disjoint” downsampled signals.

## II. Materials and Methods

### A. iEMG Dataset

This study was performed using a publicly available clinical iEMG database [45]. The data were collected from 10 healthy volunteers (six males) aged 21 to 37 years, seven myopathy subjects (five males) aged 19 to 63 years, and eight neuropathy patients (four males) aged 35 to 67 years. A standard concentric needle electrode with a leading-off area of ${0.07}{\rm mm}^2$ was inserted into brachial biceps muscles and a surface ground electrode was placed on the limb to record the iEMG signals. During data acquisition, participants applied a slight and constant contraction without needle movements. More information on the dataset is provided in Supp. Material II-A.

We constructed two datasets comprising iEMG signals recorded from the biceps brachii muscle to investigate the proposed approach:

• **Experimental dataset:** A total of 250 iEMG signals—150 signals from 10 healthy, 50 from six myopathy, and 50 from five neuropathy subjects.
• **Validation dataset:** An exclusive set (no overlap with the experimental dataset) of 100 randomly selected iEMG signals—60 signals from 10 healthy, 20 from four myopathy, and 20 from five neuropathy subjects—distributed with the same proportion across the three classes as that of the experimental dataset.

### B. iEMG Classifier Framework

**Fig. 1.**
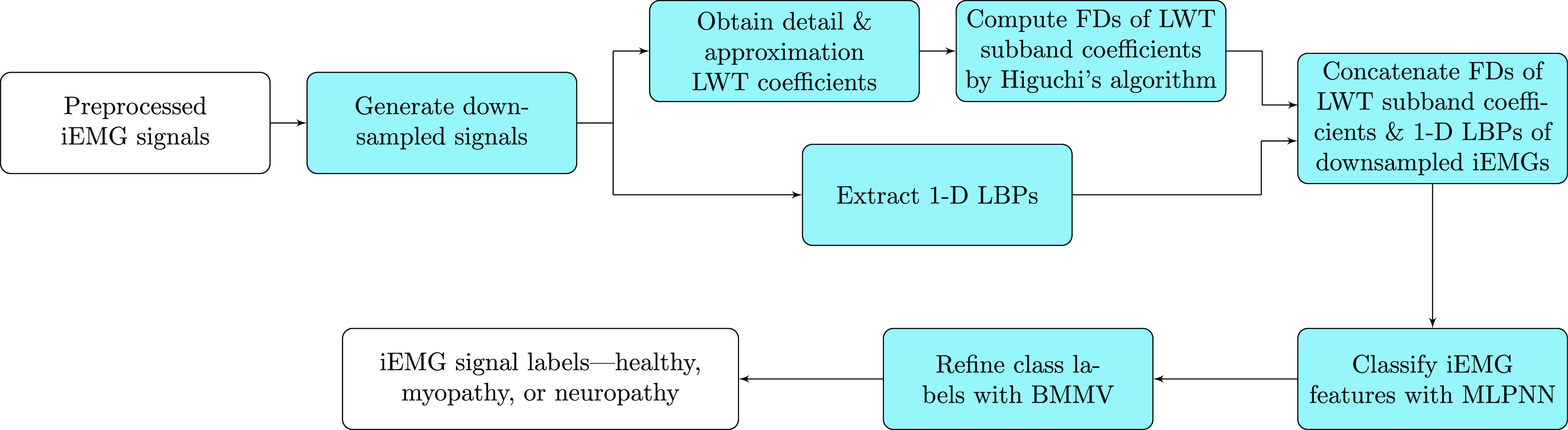
Overall schematic of MLPNN-BMMV-iEMG classifier to diagnose neuromuscular disorders. $V$ raw iEMG signals from a dataset are preprocessed and downsampled by a factor $M$ to produce $M\times V$ signals. LWT subband coefficients of each downsampled signal are fed to Higuchi's algorithm that computes the coefficients’ FDs. The 1-D LBPs of downsampled signals are deduced. The features—FDs of LWT subband coefficients and 1-D LBP—extracted from a downsampled signal are concatenated to form a feature vector. A total of $M\times V$ feature vectors are supplied to MLPNN to be classified into healthy, myopathy, or neuropathy categories. Finally, the BMMV algorithm assigns a class to an iEMG signal by applying the majority vote rule on a sequence of $M$ labels.

The MLPNN-BMMV classifier for neuromuscular disorder diagnosis is illustrated with a schematic diagram in Fig. 1. A raw iEMG signal is preprocessed and decimated by a factor of $M$ to produce a set of $M$ “disjoint” downsampled signals.^1^^1^By “disjoint” we mean that the downsampled signals are not overlapping. We remark that disjoint signals do not imply that they are statistically independent. Each downsampled iEMG signal is decomposed by LWT to generate the frequency subbands, $a[n]$ and $d[n]$, which contain the scaling/wavelet coefficients. The FDs of LWT subband coefficients are in turn computed with Higuchi's algorithm. The 1-D LBP is obtained for the downsampled iEMG signal. The fused feature set (FDs of LWT subband coefficients and 1-D LBP) from every downsampled iEMG signal is fed to the MLPNN classifier. The class labels—normal, myopathy, and neuropathy—assigned by the MLPNN are supplied to the BMMV that ultimately determines the class to which an iEMG signal belongs. The MLPNN-BMMV classifier outcome is subject to a 10-fold cross-validation (CV).

### C. Lifting Wavelet Transform

The LWT proposed by Sweldens *et. al* [46] captures the features from a nonstationary iEMG signal and localizes them in both the time and frequency domains. Compared to the discrete wavelet transform (DWT), LWT has less computational and memory requirements [47].

**Fig. 2.**
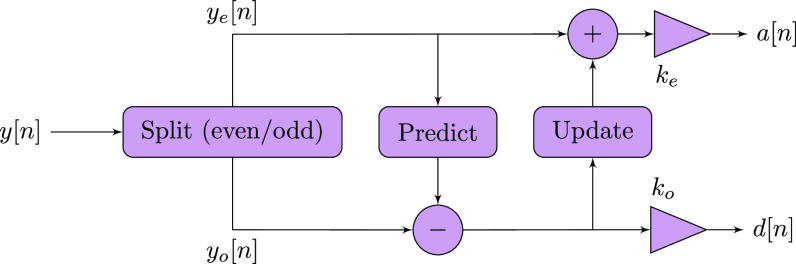
Single-level LWT scheme consisting of split, predict, update, and normalization steps. $y[n]$ denotes the input signal. $y_e[n]$ and $y_o[n]$ represent even and odd polyphase components, respectively. Predict is a high-pass filter and update is a low-pass filter. $k_e$ and $k_o$ are the normalization factors corresponding to $a[n]$ and $d[n]$, respectively.

The LWT decomposes the input signal $y[n]$ into the frequency subbands, $a[n]$ and $d[n]$, containing the scaling/wavelet coefficients. The typical lifting wavelet decomposition consists of four steps, namely, split, predict, update, and normalization [48], as depicted in Fig. 2. A detailed description of LWT is provided in Supp. Material II-B. A major advantage of the lifting scheme is that the entire information content of the input signal is preserved during the analysis process. This means that perfect reconstruction of the signal is possible by using the same predictor $P$ and updater $U$ in both the analysis and synthesis stages of the lifting scheme. Precisely, if we have $a[n]$ and $d[n]$, then the odd $y_o[n]$ and even $y_e[n]$ polyphase coefficients can be calculated, enabling the lossless reconstruction of $y[n]$.

The features extracted from the LWT coefficients in each subband represent the signal properties. The level of decomposition is selected as five as set in a DWT-based classifier proposed in [8], [31], [32]; as a result, we obtain six frequency subbands, $a_5$ and $d_1,d_2,\ldots,d_5$, containing the scaling/wavelet coefficients. Hence, six features are derived from the LWT subband coefficients. The rbio3.7 wavelet function is chosen to compute the LWT coefficients for the reasons mentioned in Supp. Material III-A.

### D. Fractal Dimension via Higuchi's Method

Various approaches have been proposed to compute the FD, e.g., Higuchi's, Katz's, and Petrosian's method [49]–[51]. FD features extracted by Katz's and Higuchi's methods were shown to be discriminative in EMG analyses [37]. Studies concerning the fractal analysis of neurological signals have demonstrated that Higuchi's method is superior to Katz's and Petrosian's methods [52], [53].

The FD computed via Higuchi's method is effectively used as a complexity measure of physiological time series, which helps identify the hidden information buried in the signal [51]. The FD estimate by Higuchi's method is claimed to be the most accurate one [36], [54]. Furthermore, for fast evaluation of signal nonlinearity, the numerical approach by Higuchi was proven to be very efficient [51]. Therefore, we rely on Higuchi's method to calculate the FDs of LWT coefficients in each subband. Interested readers are directed to Supp. Material II-C for the description of Higuchi's algorithm to compute FD [49], [55].

### E. One-Dimensional Local Binary Pattern

The 1-D LBP code represents the local activity of a signal around a data point in relation to its value [43]. It implies that LBP is not contingent on the absolute value of a signal amplitude or any DC offset. The LBP code can thus be regarded as a robust feature for iEMG classification. For details of 1-D LBP algorithm [40], one may refer to Supp. Material II-D.

### F. Feature Fusion

A feature vector of length 262 is constructed from a given iEMG or downsampled signal by concatenating the following: (i) FDs of LWT coefficients in frequency subbands, $a_5$ and $d_1,d_2,\ldots,d_5$; (ii) 1-D LBP vector of length 256.

### G. Multilayer Perceptron Neural Network

The ANN is known for its wider applicability, capability to learn complex as well as nonlinear relations, and the ability to be trained by examples. Its performance is unaffected by factors such as human fatigue, emotional states, and habituation, when used for diagnostic purposes. Moreover, ANN is well-suited for rapid identification, analysis of conditions, and real-time diagnosis [7], [8].

We implement the ANN with three layers—input, hidden, and output—which is termed as MLPNN. The features extracted from $M\times 250$ downsampled signals serve as inputs for MLPNN. The output layer consists of three nodes representing normal, myopathy, and neuropathy classes associated with the target vectors [100], [010], and [001], respectively. The three-layer MLPNN configuration with one hidden layer having 25 nodes is found suitable for our application. During the training, the initial set of random weights are adjusted with the conjugate gradient backpropagation (Polak-Ribiere update) to reduce the difference between the network outputs and the target outputs. The classifier outcomes—$M\times 250$ class labels—are supplied to BMMV, which determines the actual class of iEMG signals. We resort to a 10-fold CV to rigorously evaluate the classification performance. The $k$-fold CV diminishes the bias in classification, thereby producing a robust estimate of the classifier's error rate [7]. Moreover, the data are randomly partitioned into folds such that the classes are represented in almost the same proportion in each fold as in the entire dataset, namely, stratified $k$-fold CV, to improve the classification accuracy [7].

### H. Boyer-Moore Majority Vote Algorithm

The BMMV finds the majority of a sequence of elements using linear time and constant space [56]. For a given iEMG recording, $M$ class labels assigned by MLPNN are regarded as the elements of a sequence. The algorithm finds a majority element if there is one: that is, a class label that occurs repeatedly for more than $M/2$ times. However, if there is no majority, the algorithm will return an output label, namely, indeterminate class (see the illustration in Supp. Material II-E).

## III. Results

### A. Performance Evaluation of iEMG Classifier

The automated iEMG classifier scheme was implemented in MATLAB R2017b that assigns an iEMG signal to healthy, myopathy, or neuropathy category. 250 iEMG signals from the experimental dataset were decimated by a factor of $M=9$ (refer to Supp. Material III-B) resulting in 2250 $(9\times 250)$ downsampled signals, which were divided into a nonoverlapping training set and test set. Initially, the fused feature sets derived from the training set and the respective class labels were employed for learning. Once the MLPNN was trained, feature vectors from the test set were supplied to the classifier to determine the labels. We performed a 10-fold CV, where the dataset comprising 2250 downsampled iEMG signals was split into 10 subsets called folds, each one containing 225 signals. At every instance, 225 iEMG signals in a fold were used for testing and the remaining 2025 signals from the other nine folds for training. This procedure was repeated 10 times by changing the training and the corresponding test set. After the 10-fold CV, BMMV assigned a class label to each iEMG signal by applying the majority rule on the sequence of the respective downsampled signal labels.

**Fig. 3.**
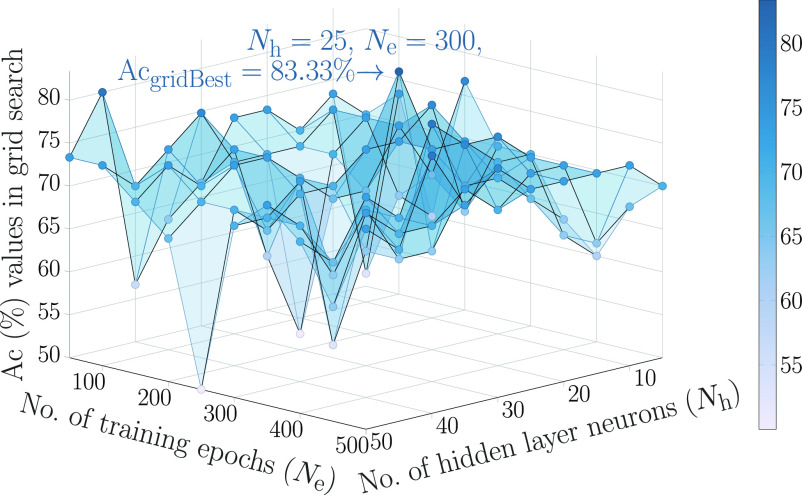
Graphical representation of the 3-D landscape of $\mathrm{Ac}$ values returned by a grid search with a 70–30 hold-out validation for tuning the MLPNN hyperparameters, $N_{\mathrm{h}}$ and $N_{\mathrm{e}}$. To estimate the optimal set of hyperparameters, $N_{\mathrm{h}}$ and $N_{\mathrm{e}}$ were varied within the interval $[5,\,50]$ in steps of five and within $[50,\,500]$ in steps of 50, respectively. The hyperparameter values, $N_{\mathrm{h}}=25$ and $N_{\mathrm{e}}=300$, corresponding to $\mathrm{Ac}_{\mathrm{gridBest}}$ denoted by the tallest peak marking an accuracy of 83.33% in the surface plot are adopted in our implementation.

The hyperparameters of MLPNN—number of hidden layer neurons $N_{\mathrm{h}}$ and total training epochs $N_{\mathrm{e}}$—were estimated to be 25 and 300, respectively, with a 70–30 hold-out validation performed on a grid of possible values as illustrated in Fig. 3 using the validation set.

The performance evaluation measures, namely, total classification accuracy $\mathrm{Ac}$, sensitivity $\mathrm{Se}$, and specificity $\mathrm{Sp}$, are indicative of the effectiveness of the classifier. We compare the performance of our approach with state-of-the-art approaches using these measures defined as follows:

\begin{align*}
\mathrm{Ac}&:= \frac{\text{No. of correctly classified iEMG signals}}{\text{Total iEMG signals}}\times 100\%\tag{1}
\\
\mathrm{Se}&:= \frac{\text{TP}}{\text{(TP + FN)}}\times 100\%\tag{2}
\\
\mathrm{Sp}&:= \frac{\text{TN}}{\text{(TN + FP)}}\times 100\% \tag{3}
\end{align*}

where TP, TN, FP, and FN refer to the number of true positive, true negative, false positive, and false negative instances with respect to the class under consideration, respectively [26]. In (1)–(3), true or false denotes the assigned classification being correct or incorrect, while positive or negative denotes the assignment to the positive or the negative category.

The proposed MLPNN-BMMV and subcategories of the state-of-the-art classifiers—discriminant analysis, decision tree, ensemble learning, KNN, and SVM—were rigorously validated by executing them 25 times to perform iEMG classification with a 10-fold CV. The investigated classifiers were supplied with the fused feature sets deduced from the experimental dataset. The average $\mathrm{Ac}$ computed from 25 runs is recorded in Table I for every approach to highlight the improvement in classification ($\mathrm{Ac}=99.87\%$) achieved by MLPNN-BMMV. We listed other performance measures of the best-performing classifier in each category, i.e., linear discriminant analysis (LDA), fine tree (FT), ensemble subspace discriminant (ESD), fine KNN (FKNN), and quadratic SVM (QSVM), besides those of MLPNN-BMMV in Table II. Notice that the performance of MLPNN, QSVM, and cubic SVM (CSVM) is comparable and better than the rest of the classifiers. Recall that the improvement in classification is not contingent only on the choice of the classifier, rather heavily relies on the downsampling since “artificially” increasing the sample size reduces the variance of the estimator (refer to the last two rows of Table I).

**TABLE II:** Average Performance Measures (%) in (1)–(3) for the Best iEMG Classifier Under Each Category. Each Classifier Performed a 10-Fold CV 25 Times

Performance	LDA	FT	ESD	FKNN	QSVM	MLPNN-BMMV
Measures (%)						
$\mathrm{Ac}$	50.40	73.20	78.67	74.80	85.97	99.87
$\mathrm{Se\,(nor)}$	50.00	78.00	85.12	82.00	90.48	99.97
$\mathrm{Se\,(myo)}$	56.00	64.00	72.64	56.00	80.00	99.68
$\mathrm{Se\,(neuro)}$	46.00	68.00	65.36	72.00	78.40	99.76
$\mathrm{Sp\,(nor)}$	68.00	75.00	73.80	68.00	84.96	99.72
$\mathrm{Sp\,(myo)}$	73.50	89.00	93.94	90.00	94.98	99.98
$\mathrm{Sp\,(neuro)}$	80.50	90.00	92.50	94.50	95.00	99.96

The average confusion matrix is shown in Table III to provide empirical evidence for the outstanding performance of the approach. We remark that the classification performance did not alter significantly, when we repeated the experiment with a 4-fold CV, suggesting that the improvement in the results is not due to overfitting. Each column and row of the confusion matrix $\mathbf {C}$ denote the percentage of cases in the desired and the actual class, respectively, or vice versa as given below:

\begin{equation*}
\mathbf {C}= \left[ {\begin{array}{ccc}C_{\mathrm{HH}} & C_{\mathrm{HM}} & C_{\mathrm{HN}}\\
C_{\mathrm{MH}} & C_{\mathrm{MM}} & C_{\mathrm{MN}}\\
C_{\mathrm{NH}} & C_{\mathrm{NM}} & C_{\mathrm{NN}}\\
\end{array}} \right]
\end{equation*}

where the subscripts $\mathrm{H}$, $\mathrm{M}$, and $\mathrm{N}$ represent the healthy, myopathy, and neuropathy classes, respectively. For instance, $C_{\mathrm{HM}}$ is calculated as follows:

\begin{equation*}
C_{\mathrm{HM}}= \frac{\text{No. of class H signals classified as class M}}{\text{Total signals classified as class M}}\times 100\%.
\end{equation*}

Thus, the percentages of correctly classified signals are located along the main diagonal of $\mathbf {C}$ and the nondiagonal values denote the misclassification percentages.

**TABLE III:** Average Confusion Matrix Constructed With the MLPNN-BMMV (10-Fold CV) Classifier Outcome (%). The Classifier Was Executed 25 Times With the Experimental Dataset

Class Label	Healthy	Myopathy	Neuropathy
Healthy	99.97	0	0.03
Myopathy*	0.24	99.68	0
Neuropathy**	0.16	0	99.76

^*^indeterminate class – 0.08% ^**^indeterminate class – 0.08%.

Most importantly, Table IV summarizes the comparison of performance measures between our method and those reported in the literature using the same iEMG dataset. The last row of Table IV corresponding to MLPNN-BMMV underscores the surpassing performance of the proposed classifier in terms of $\mathrm{Ac}$, $\mathrm{Se}$, and $\mathrm{Sp}$. To conclude, the classification results from MLPNN-BMMV are quite reliable to aid the diagnosis of neurogenic and myogenic disorders, which is empirically verified by the performance measures (%): $\mathrm{Ac}=99.87\pm 0.25$, $\mathrm{Se\,(nor)}=99.97\pm 0.13$, $\mathrm{Se\,(myo)}=99.68\pm 0.95$, $\mathrm{Se\,(neuro)}=99.76\pm 0.66$, $\mathrm{Sp\,(nor)}=99.72\pm 0.61$, $\mathrm{Sp\,(myo)}=99.98\pm 0.10$, and $\mathrm{Sp\,(neuro)}=99.96\pm 0.14$. In addition, we have listed the classification performance reported by a few binary classifiers (Nor and ALS) with the same iEMG dataset in Supp. Material III-C for comparison.

**TABLE IV:** Comparison of Performance Measures (%) of the Proposed (Boldfaced) and State-of-the-Art iEMG Classifiers. Nor, Myo, and Neuro (ALS) Represent Normal, Myopathy, and Neuropathy (Amyotrophic Lateral Sclerosis), Respectively. All Approaches Classified the iEMG Data From the Same Dataset

Method (Year)	Classes/Study Group	$\mathrm{Ac}$	$\mathrm{Se\, (nor)}$	$\mathrm{Se\, (myo)}$	$\mathrm{Se\, (neuro)}$	$\mathrm{Sp\, (nor)}$	$\mathrm{Sp\, (myo)}$	$\mathrm{Sp\, (neuro)}$
[26] (2013)	3/Nor, Myo, Neuro	97.00	98.70	95.30	96.00	95.60	97.60	97.50
[15] (2014)	3/Nor, Myo, ALS	92.55	90.30	91.00	96.33	94.83	97.16	96.83
[57] (2014)	3/Nor, Myo, ALS	93.08	99.00	93.25	87.00	96.62	93.00	100.00
[34] (2015)	3/Nor, Myo, ALS	96.67	94.75	95.66	99.58	97.83	98.00	99.17
[5] (2015)	3/Nor, Myo, ALS	98.00	100.00	94.00	96.00	97.00	99.50	99.50
[19] (2015)	3/Nor, Myo, ALS	84.00	89.33	64.00	88.00	–	–	–
[18] (2016)	3/Nor, Myo, ALS	88.60	88.00	91.00	93.00	–	–	–
[21] (2019)	3/Nor, Myo, ALS	99.03	99.58	98.50	97.59	–	–	–
**MLPNN-BMMV**	3/Nor, Myo, Neuro	${\bf 99.87}$	${\bf 99.97}$	${\bf 99.68}$	${\bf 99.76}$	${\bf 99.72}$	${\bf 99.98}$	${\bf 99.96}$

In proportion to the signals available under each category in the iEMG dataset, we selected an imbalanced dataset (150 normal, 50 myopathy, and 50 neuropathy). Subsequently, we also performed the classification of a balanced dataset, i.e., 50 iEMG signals under each category, using MLPNN-BMMV with a 10-fold CV 25 times. To this end, 50 normal iEMGs were uniform-randomly selected from the available set of 150 signals acquired from healthy subjects. The resulting performance measures (%) are almost similar to those obtained earlier, which are listed below: $\mathrm{Ac}=99.97\pm 0.13$, $\mathrm{Se\,(nor)}=100.00$, $\mathrm{Se\,(myo)}=99.92\pm 0.40$, $\mathrm{Se\,(neuro)}=100.00$, $\mathrm{Sp\,(nor)}=99.96\pm 0.20$, $\mathrm{Sp\,(myo)}=100.00$, and $\mathrm{Sp\,(neuro)}=100.00$.

This application in MATLAB runs on a Microsoft Windows 10 Home Single Language HP notebook (Intel(R) Core(TM) i5-6200U 2.30 GHz CPU, 4.0 GB 2401 MHz RAM, 64-bit Operating System). The average execution time taken by MLPNN-BMMV to complete 25 trial runs with a 4-fold and 10-fold CV is $18.02\pm 2.70$ s and $48.82\pm 5.15$ s, respectively.

### B. Limitations of the Study

Admittedly, the study has two inherent limitations owing to the iEMG dataset, even though several researchers have employed this dataset for validating their classification algorithms. (1) **Lack of Age-Matched Groups**. A one-way-between-subjects analysis of variance (ANOVA) was conducted to compare the age across the three groups. The analysis shows that there is a significant difference in the age across groups [$F(2,22)=19.80$, $p< 0.001$]. The post hoc comparisons using the Tukey's honest significance test reveal that the age in the healthy group ($27.20\pm 4.54$) is significantly lower ($p< 0.001$) than the neuropathy group ($56.50\pm 9.98$), but not than the myopathy group ($36.28\pm 14.60$, $p=0.17$). Moreover, the age in the neuropathy group is significantly different from the myopathy group ($p=0.001$). (2) **Small Sample Size**. Even though we considered 250 samples in the dataset, the iEMG signals were recorded from 25 subjects—10 healthy, seven myopathy, and eight neuropathy subjects—and hence classifiers would encounter the problem of small sample size. Strictly speaking, this challenge is difficult to overcome in most instances: small sample size is prevalent in medical/biological datasets, as it is hard to acquire a huge number of samples; besides, the bias is evident even with a sample size of 1000 in the performance estimates obtained from $k$-fold CV [58]. We attempted to circumvent this shortcoming empirically by changing the $k$ value in the CV ($k=4,10$) and by performing repeated CV (25 times). The fact that the estimates from these approaches (changing $k$ and repeated CV) have a small variance is a good indicator to ensure the quality of the results. Another expedient to obtain robust and unbiased estimates with a small sample size is by resorting to a nested CV recommended by Varma *et al* in [59], which we will investigate in the future.

## IV. Conclusion

Over the past two decades, the classification of iEMG signals into healthy, myopathy, and neuropathy categories has been an effervescent research topic, as it could potentially assist the diagnosis of neuromuscular disorders [5], [7], [8], [11], [13], [15], [16], [21], [26]–[34], [60]. In order to enhance the performance of an iEMG classifier, the extraction of an efficient feature set is deemed crucial [8], [9]. Therefore, research efforts have been dedicated to achieving a higher classification performance by experimenting with diverse sets of features that could maximize the discriminant power with reduced redundancy.

The proposed classifier makes use of fused feature sets, which comprise FDs of LWT subband coefficients from level-five decomposition and 1-D LBP of decimated iEMG signals. The MLPNN implemented with the selected hyperparameters, $N_{\mathrm{h}}=25$ and $N_{\mathrm{e}}=300$, returns a sequence of class labels for each signal. Subsequently, BMMV assigns a class label to every iEMG signal by applying the majority vote rule to the sequence of labels. Notably, as reported in Section III-A, the classification performance measures of MLPNN-BMMV outclassed the state-of-the-art approaches tested with the same dataset.

We envisage the following future research directions: (i) The performance of MLPNN-BMMV will be tested using the nested CV as mentioned in Section III-B. By rigorously validating our approach with iEMG datasets having age-matched groups and larger sample sizes, an affordable expert system will be designed for real-time applications in clinical practice. (ii) This classification framework will be applied to sEMG/high-density sEMG data to investigate whether it requires to be modified to classify noninvasively recorded EMG data into the underlying categories. (iii) The proposed approach will be tailored to analyze other electrophysiological signals: diagnosis of sleep and emotion disorders from EEG and ECG; predicting the risk of preterm labor from electrohysterogram.

## Supplementary Materials

Additional information related to the following sections can be found in the downloadable Supp. Material file: Section I, II-A, II-C, II-D, II-E, II-H, and III-A. Furthermore, the rationale for the choice of rbio3.7 wavelet for performing LWT and the empirical procedure to determine the decimation factor for decomposing the iEMG signals are described. A multimedia file pertaining to this article can be downloaded and viewed from the following link: https://tinyurl.com/iEMG-NMD-Detection.


