# Supplementary material for: Robust Classification of Intramuscular EMG Signals to Aid the Diagnosis of Neuromuscular Disorders
Source: IEEE Open J Eng Med Biol. 2020 Aug 17;1:235–42. doi: 10.1109/OJEMB.2020.3017130 (PMC8975248; doi:10.1109/OJEMB.2020.3017130)
Supplement: Supplementary file 1 [file supp1-3017130.pdf]

Supplementary Materials

Robust Classification of Intramuscular EMG Signals to Aid the Diagnosis of Neuromuscular Disorders

Shobha Jose, S. Thomas George, M. S. P. Subathra, Vikram Shenoy Handiru, *Member, IEEE*, Poornaselvan Kittu Jeevanandam, Umberto Amato, and Easter S. Suviseshamuthu, *Member, IEEE*

I. Introduction

A. iEMG Signal Changes in Neuromuscular Disorder

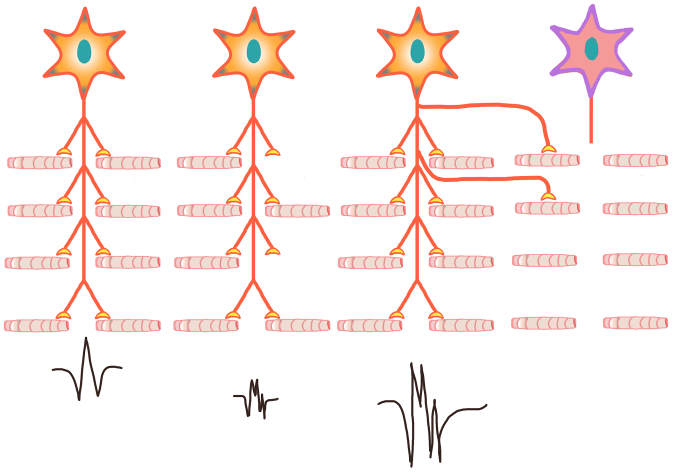

Fig. 1. Left: MUAPs of healthy individuals have two to four phases. Middle: In myopathy patients, the number of functional muscle fibers per MU decreases, leading to short-duration, small-amplitude, and polyphasic MUAPs. Right: In neuropathy patients, the number of muscle fibers in the MU increases, which causes long-duration, high-amplitude, and polyphasic MUAPs [1].

II. Materials and Methods

A. iEMG Dataset

The iEMG recording was monitored visually and audibly by means of a computer monitor and a speaker. The amplitude and duration of an MUAP captured by the needle electrode were measured on an oscilloscope panel. An MUAP was selected if it was more than a threshold and generated by a single MU. No more than three different MUAPs were recorded at a single insertion site within an epoch of 50 ms. The signals, which were not too complex or noisy and thus suitable for evaluating MUAP parameters, were recorded for a period of 11.2 s. Care was taken to record only once from an MU and to ensure that the entire muscle was explored. The recorded iEMG signals were amplified 4000 times with a differential amplifier and filtered by an analog band-pass filter with a lower and upper cut-off frequency of 2 Hz and 10 kHz, respectively. The signals were then sampled at a rate of 23 438 Hz and digitized with a 16-bit resolution.

B. Lifting Wavelet Transform

1) **Split or lazy wavelet transform**. This step simply splits the input signal $y[n]$ into two groups—one consisting of even polyphase coefficients $y_e[n]$ and the other comprising odd polyphase coefficients $y_o[n]$, where $y_e[n] = y[2n]$ and $y_o[n] = y[2n+1]$. Thus, splitting the input signal into odd and even polyphase coefficients involves no complex mathematical operation, and hence it is termed as the lazy wavelet transform.

2) **Predict or dual lifting**. This step is intended for the prediction of one of these two disjoint groups from the other. For example, one can predict $y_o[n]$ coefficients by a linear combination of their l neighboring $y_e[n]$ coefficients, in which case the predictor for each $y_o[n]$ is given by:

$$P(y_e)[n] = \sum_l p_l y_e[n+l]$$

where p_l's are scalars. The prediction residual is therefore expressed as

$$d[n] = y_o[n] - P(y_e)[n].$$

Given $y_e[n]$ and $d[n]$, it is straightforward to recover $y_o[n]$ as follows:

$$y_o[n] = d[n] + P(y_e)[n]. \tag{1}$$

This prediction procedure is equivalent to applying a high-pass filter to $y[n]$. Thus, by replacing $y_o[n]$ with (1), a new representation can be obtained for $y[n]$, which contains the same information as the original signal $y[n]$. Note that for an underlying locally smooth signal, $d[n]$ will be negligible.

3) **Update or primal lifting**. This step transforms $y_e[n]$ into a low-pass filtered and subsampled version of $y[n]$. The coarse approximation results in $a[n]$ by updating $y_e[n]$ with a linear combination of $d[n]$:

$$a[n] = y_e[n] + U(d)[n]$$

where $U(d)[n]$ is a linear combination of l neighboring d values

$$U(d)[n] = \sum_l u_l d[n+l]$$

with u_l's being scalars. Given $d[n]$ and $a[n]$, we can obtain $y_e[n]$ as given below:

$$y_e[n] = a[n] - U(d)[n]. \tag{2}$$

4) **Normalization or scaling**. The outputs of the lifting scheme are weighted by the factors k_e and k_o, where k_e is the inverse of k_o. These values are used to normalize the energy of the scaling and wavelet functions, respectively.

C. Fractal Dimension via Higuchi's Method

Higuchi's method is employed to compute the FD of LWT coefficients in each subband as follows [2], [3]. Consider a time series, $x[n]$, $n = 1,2,\ldots,N$. In this study, $x[n]$ refers to LWT coefficients contained in each frequency subband, $a_5, d_1, d_2, \ldots, d_5$, from level-five decomposition.

1. Construct k new times series x_o^k defined as:

$$x_o^k = \{x[o], x[o+k], x[o+2k], \ldots, x[o + \lfloor \frac{N-o}{k} \rfloor k]\}$$

where $o = 1,2,\ldots,k$ denotes the initial time value, k represents the discrete time interval between points, and $\lfloor \cdot \rfloor$ returns the integer part of the argument.

2. Compute the average length of each time series x_o^k as given by:

$$L_o(k) := \frac{(N-1)}{k} \frac{\sum_{j=1}^{\lfloor \frac{N-o}{k} \rfloor} |x[o+jk] - x[o+(j-1)k]|}{\lfloor \frac{N-o}{k} \rfloor k}$$

where $(N-1)/\lfloor (N-o)/k \rfloor k$ is a normalization factor.

3. Calculate the average length of all x_o^k's having the same delay k as:

$$L(k) = \sum_{o=1}^{k} L_o(k).$$

4. Estimate FD (denoted as D) to be the slope of the least square linear fit of the curve, $\ln(L(k))$ versus $\ln(1/k)$, which implies that $L(k) \propto k^{-D}$. Therefore, when $L(k)$ is plotted against $1/k$, with $k = 1,2,\ldots,k_{\max}$, on a double logarithmic scale, the data must remain on a straight line with a slope equal to D.

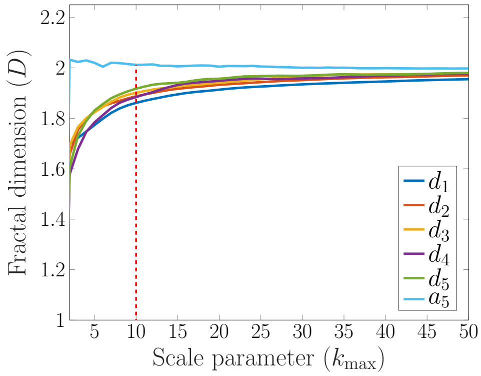

Fig. 2. For the LWT coefficients in each subband from level-five decomposition, D was computed with Higuchi's method by selecting an integer value for $k_{\max}$ in the interval $[2,50]$. Around $k_{\max} = 10$, D plateaus meaning that there is no benefit from further calculations, i.e., computing D with $k_{\max} > 10$.

In order to select a suitable value for the scaling parameter $k_{\max}$, D values computed for the wavelet subband coefficients were plotted against $k_{\max} \in \{2,3,\ldots,50\}$. The value of $k_{\max}$ for which D reaches a plateau is regarded as the saturation point as suggested in [4], [5]. In our case, $k_{\max}$ was selected as 10 as shown in Fig. 2.

D. One-Dimensional Local Binary Pattern

For a given data point $s[n]$ in a signal, the 1-D LBP operator is defined as an ordered set of binary comparisons between $s[n]$ and its neighboring $S/2$ data points occurring before and after $s[n]$. For our application, four neighboring data points are considered before $(s[1],\ldots,s[4])$ and after $(s[5],\ldots,s[8])$ each data point $s[n]$. The 1-D LBP code is constructed for $s[n]$ by comparing its value with each one of its neighboring data points, $S = \{s[1], s[2], \ldots, s[8]\}$, and thresholding the respective differences, $\Gamma(s[k] - s[n])$, $k = 1,2,\ldots,\mathrm{card}(S)$, to produce a set of binary numbers

$$\Gamma(s[k]-s[n]) = \begin{cases} 1, s[k]-s[n] \geq 0 \\ 0, s[k]-s[n] < 0. \end{cases}$$

Thus, an eight-digit binary code is obtained for a neighborhood S surrounding a data point $s[n]$. The decimal equivalent of the binary code ranging between 0 and 255 is then obtained as given by

$$\mathrm{LBP}(s[n]) = \sum_{k=1}^{\mathrm{card}(S)} \Gamma(s[k]-s[n])\, 2^k \tag{3}$$

where $\mathrm{card}(\cdot)$ is the cardinality of the set in the argument. The LBP code in (3) represents the local structural information around $s[n]$. After sequentially performing this operation for all the data points in a signal, a histogram is constructed with the decimal equivalents of the LBP codes, which serves as the LBP feature for our iEMG classification task.

E. Boyer-Moore Majority Vote Algorithm

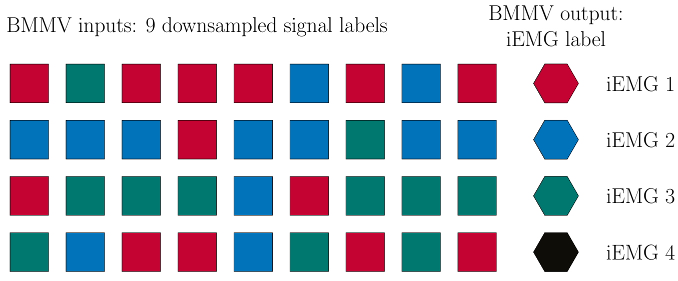

Fig. 3. An illustration to demonstrate the class assignment by the BMMV algorithm. Four iEMG recordings are arranged along the rows, each of which is downsampled by a factor nine, i.e., $M = 9$. Each row of colored squares (left) represents a set of nine class labels corresponding to "disjoint" downsampled signals as determined by the MLPNN. The colored hexagon in the respective row (right) denotes the class assigned by the BMMV to an iEMG datum based on the majority vote rule. The filled-in colors imply the following categories: red ⇒ healthy, green ⇒ myopathy, blue ⇒ neuropathy, and black ⇒ indeterminate.

III. Results

A. Choice of Wavelet Function for LWT

Since TF characteristics of wavelet functions vary, it is imperative to select an appropriate wavelet function to perform LWT. This means that wavelet coefficients reflect the degree of similarity between the wavelet function and the decomposed iEMG signals, i.e., larger wavelet coefficients imply a higher degree of similarity [6]. The downsampled iEMG signals are nonstationary and nondecaying as illustrated in Fig. 4. The mother wavelets in Fig. 5, namely, db4, sym5, coif2, bior6.8,

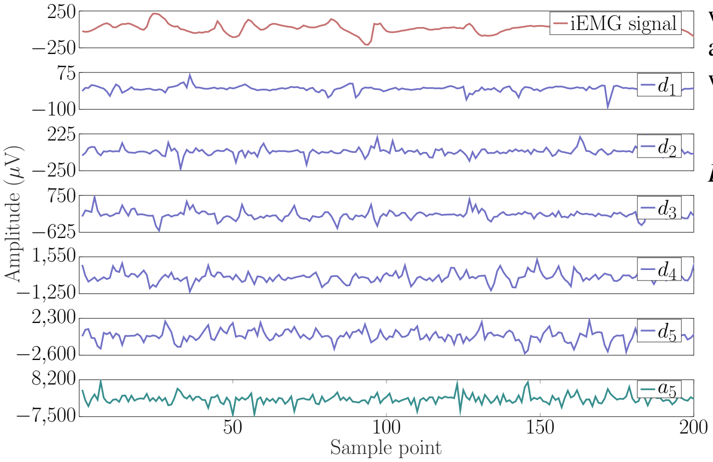

Fig. 4. A section of a downsampled iEMG recording (first row). The frequency subbands, $a_5, d_1, d_2, \ldots, d_5$, from level-five LWT decomposition of the downsampled iEMG signal contain scaling/wavelet coefficients (rows two to seven). From the shape of the signal in the first row, one can infer that the downsampled iEMG signals are nonstationary and nondecaying.

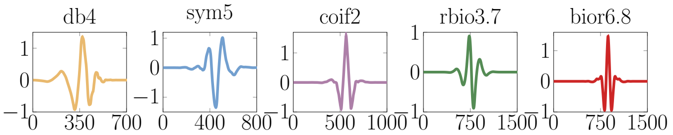

Fig. 5. Mother wavelet functions, which have shapes somewhat similar to iEMG signals that are nonstationary and nondecaying as shown in Fig. 4.

and rbio3.7, were hence selected because of their similarity to iEMG signals.

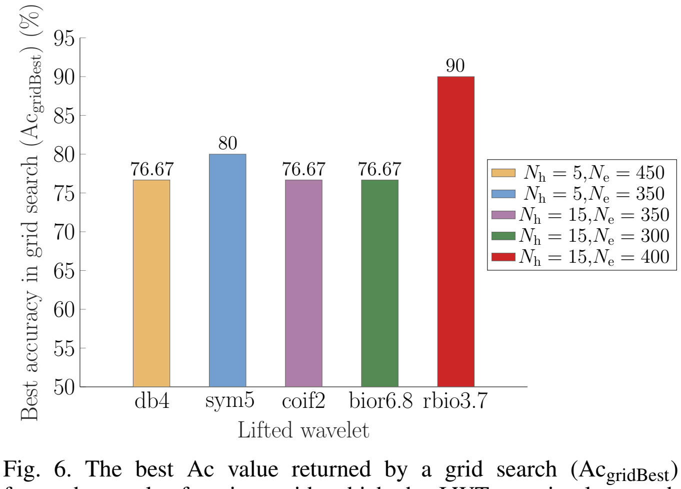

Fig. 6. The best Ac value returned by a grid search ($\mathrm{Ac}_{\mathrm{gridBest}}$) for each wavelet function, with which the LWT was implemented, is shown; the associated hyperparameter values are displayed in the right panel. Note that rbio3.7 wavelet yielded the maximum $\mathrm{Ac}_{\mathrm{gridBest}}$ (90%), and hence we selected rbio3.7 for our implementation.

To further narrow down the choice, fused feature sets—FDs of LWT subband coefficients computed with a chosen mother wavelet and 1-D LBP—from the validation iEMG dataset were supplied to MLPNN. For each wavelet function, the best accuracy achieved by the classifier ($\mathrm{Ac}_{\mathrm{gridBest}}$) via a grid search was noted. Among the $\mathrm{Ac}_{\mathrm{gridBest}}$ values, the maximum was attained by rbio3.7 wavelet function (see Fig. 6), and therefore we chose rbio3.7 wavelet for our classifier framework.

B. *Determining Decimation Factor*

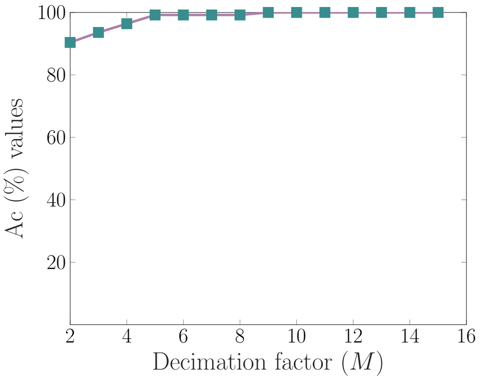

Fig. 7. The Ac (%) values of MLPNN-BMMV classifier supplied with features extracted from iEMG signals in the experimental dataset, when the signals were downsampled by integer values in the interval [2, 15]. Notice that for $M \geq 5$, the classifier performance (in terms of Ac) remains nearly consistent.

The decimation factor M was determined to be nine by the following empirical procedure. The iEMG signals from the validation dataset were downsampled by an integer factor, which was varied from two to 15 in steps of one in every instance. The fused feature sets constructed from the downsampled signals were fed to MLPNN performing 70–30 hold-out validation. The hyperparameter values were varied in a grid search. The $\mathrm{Ac}_{\mathrm{gridBest}}$ values were noted for M values in the interval [2, 15], whose maximum was attained for $M = 9$.

To evaluate how much the performance of MLPNN-BMMV classifier (particularly the BMMV algorithm) is reliant on the choice of M, we conducted the following investigation. The iEMG signals in the experimental dataset were decimated by the integer values in the range [2, 15]. For each M value, the features extracted from the downsampled signals were classified using the MLPNN-BMMV classifier (10-fold CV) and the respective Ac (%) was recorded. The empirical results are plotted in Fig. 7, which show that the BMMV-based classifier performance is almost insensitive to the choice of $M \geq 5$.

C. *Performance Comparison of Binary iEMG Classifiers*

The following studies have reported the classification performance measures, i.e., Ac, Se, and Sp, obtained from a relatively simple binary classification[1]—healthy or ALS—using the same iEMG dataset as ours. Interestingly, the MLPNN-BMMV approach resulted in a higher Ac value (99.87%) than those listed in Table I.

[1]Multi-class classification is often deemed more difficult than the binary classification [7].

TABLE I. Performance measures (%) of iEMG classifiers reported in the literature, which performed binary classification of iEMG data from the dataset we used to validate our approach. Nor and ALS represent normal and amyotrophic lateral sclerosis, respectively.

Method (Year)	Classes/Study Group	Ac	Se	Sp
[8] (2014)	2/Nor, ALS	92.50	98.00	76.00
[9] (2016)	2/Nor, ALS	95.00	92.54	93.00
[10] (2017)	2/Nor, ALS	96.69	97.59	94.24
[11] (2017)	2/Nor, ALS	96.80	98.80	94.80

References

[1] D. C. Preston and B. E. Shapiro, *Electromyography and Neuromuscular Disorders E-Book: Clinical-Electrophysiologic Correlations (Expert Consult-Online and Print).* Elsevier Health Sciences, 2012.

[2] T. Higuchi, "Approach to an irregular time series on the basis of the fractal theory," *Physica D: Nonlinear Phenomena*, vol. 31, no. 2, pp. 277–283, 1988.

[3] R. Esteller, G. Vachtsevanos, J. Echauz, and B. Litt, "A comparison of waveform fractal dimension algorithms," *IEEE Transactions on Circuits and Systems I: Fundamental Theory and Applications*, vol. 48, no. 2, pp. 177–183, 2001.

[4] W. Klonowski, E. Olejarczyk, and R. Stepien, "'Epileptic seizures' in economic organism," *Physica A: Statistical Mechanics and its Applications*, vol. 342, no. 3-4, pp. 701–707, 2004.

[5] C. Gómez, Á. Mediavilla, R. Hornero, D. Abásolo, and A. Fernández, "Use of the Higuchi's fractal dimension for the analysis of MEG recordings from Alzheimer's disease patients," *Medical Engineering & Physics*, vol. 31, no. 3, pp. 306–313, 2009.

[6] F. Duan, L. Dai, W. Chang, Z. Chen, C. Zhu, and W. Li, "sEMG-based identification of hand motion commands using wavelet neural network combined with discrete wavelet transform," *IEEE Transactions on Industrial Electronics*, vol. 63, no. 3, pp. 1923–1934, 2015.

[7] Z. Noumir, P. Honeine, and C. Richard, "Multi-class least squares classification at binary-classification complexity," in *Proceedings of IEEE Statistical Signal Processing Workshop (SSP)*, 2011, pp. 277–280.

[8] A. B. M. S. U. Doulah and S. A. Fattah, "Neuromuscular disease classification based on mel frequency cepstrum of motor unit action potential," in *Proceedings of International Conference on Electrical Engineering and Information & Communication Technology*, 2014, pp. 1–4.

[9] V. K. Mishra, V. Bajaj, A. Kumar, and G. K. Singh, "Analysis of ALS and normal EMG signals based on empirical mode decomposition," *IET Science, Measurement & Technology*, vol. 10, no. 8, pp. 963–971, 2016.

[10] A. Sengur, M. Gedikpinar, Y. Akbulut, E. Deniz, V. Bajaj, and Y. Guo, "DeepEMGNet: An application for efficient discrimination of ALS and normal EMG signals," in *Proceedings of International Conference Mechatronics*, 2017, pp. 619–625.

[11] A. Sengur, Y. Akbulut, Y. Guo, and V. Bajaj, "Classification of amyotrophic lateral sclerosis disease based on convolutional neural network and reinforcement sample learning algorithm," *Health Information Science and Systems*, vol. 5, no. 1, p. 9, 2017.
